# Mea Culpa! The Role of Guilt in the Work-Life Interface and Satisfaction of Women Entrepreneur

**DOI:** 10.3390/ijerph191710781

**Published:** 2022-08-30

**Authors:** Silvia De Simone, Jessica Pileri, Marina Mondo, Max Rapp-Ricciardi, Barbara Barbieri

**Affiliations:** 1 Department of Pedagogy, Psychology and Philosophy, University of Cagliari, 09123 Cagliari, Italy; 2 Department of Dynamic Clinical Psychology and Health Studies, Sapienza University of Rome, 00185 Roma, Italy; 3 Department of Psychology, University of Gothenburg, 40530 Gothenburg, Sweden; 4 Department of Political and Social Sciences, University of Cagliari, 09124 Cagliari, Italy

**Keywords:** life satisfaction, job satisfaction, guilt, conflict, enrichment, women entrepreneurs

## Abstract

The purpose of this study is to analyze the role of mediator of Guilt (in both directions: Family Interference with Work (FIW) and Work Interference with Family (WIF)) in the relationship between Conflict, Job and Life Satisfaction, also investigating the role of Enrichment as moderator. Using PROCESS Macro, the hypothesized models are tested on a sample of 161 women entrepreneurs. Both the mediating role of guilt and the moderating role of enrichment were analyzed through models of mediation and moderate mediation. Results from the analysis support the hypothesized models. Guilt FIW and Guilt WIF mediate the relationship between work Conflict and Job satisfaction, as well the relationship between Conflict and Life satisfaction, and at the same time, Enrichment moderated the mediating processes by which the Conflict affects Job and Life satisfaction via Guilt FIW and Guilt WIF. This study is one of the few that takes into consideration both Conflict and Enrichment in a sample of women entrepreneurs and examines Guilt, which many times presents itself as an “invisible” factor in studies on the work–family interface.

## 1. Introduction

There is a growing number of women entrepreneurs all over the world, as well as in Italy, which gives us the context of our study [1]. High levels of life satisfaction are important for human functioning [2], and in the same way, job satisfaction is an indispensable element for the well-being of people. Nevertheless, the drivers of the life satisfaction of entrepreneurs are still unclear [3,4]. It is known that a satisfying life has a positive effect on both productivity [5], including for entrepreneurs [6], and job satisfaction [7]. Research states that flexibility and freedom to manage working hours do not apply to typically female activities [8]. For this reason, we believe that it is essential to consider the work–family interface when investigating the life satisfaction and job satisfaction of female entrepreneurs. The work–family conflict has not been extensively studied in relation to entrepreneurs [9]. The literature has studied the work–family conflict of workers for a long time, excluding entrepreneurs, perhaps thinking that they have greater working flexibility that allows them to better balance work and family [10]. This is surprising because being an entrepreneur means having job responsibilities that can burden family life. Being an entrepreneur generates higher costs, especially in terms of both professional and personal pressures compared to of employees. In the same way, it is surprising that there is little attention paid to women entrepreneurs [11] in relation to the work–family interface. In fact, women experience higher levels of work–family conflict than men [12,13,14,15]. This trend is even greater when it comes to women entrepreneurs [16]. Indeed, women entrepreneurs can have multiple roles both in the family and in the company. Such roles, when poorly managed, can cause conflicts. In general, compared to an employed woman, a woman entrepreneur has more responsibilities and works more hours, with more difficulties in balancing life and work [17,18]. Despite the difficulties faced by women and women entrepreneurs, we also examine the possible benefits balancing professional and family roles. In fact, as already stated, several studies show the social and psychological resources acquired by participating in multiple roles in life [19]. Many scholars have recognized that the family plays a key role in the entrepreneurial experience [9,20,21,22,23]. For example, Rogoff and Heck [24] said that the role of the family can “*feed the fire of entrepreneurship*”. Because of these observations, we believe that it is more appropriate to deal with the work–family interface considering both Conflict and Enrichment, especially for women. In fact, using this interactive model ensures that both environments (family and work) and their relationships are recognized by providing more information on how women balance both domains [25,26]. We also believe that both processes can act simultaneously on both business and family life [19,27]. To this purpose, we add another element often overlooked in the investigation of the work–family interface: guilt. According to Wharton and Erickson [28], we believe that although research on the relationships between work and family considers multiple roles, it often ignores the emotional component. It is the individual who influences one’s own experience of role conflict or enrichment [29]. Guilt is probably one of the strategies used by women to manage role conflicts [30]. Furthermore, guilt has been taken into account only as an invisible factor [31] in quantitative analysis on women entrepreneurs despite its emergence in some qualitative research [32,33,34]. To the best of our knowledge, there are no empirical studies on Guilt in women entrepreneurs. So, this paper is among the few studies that: (a) considers both “Conflict and “Enrichment in a sample of women entrepreneurs; (b) examines the family and work domains in terms of guilt, which many times presents itself as an “invisible” factor in studies on the work–family interface [31]; and (c) considers essential outcomes for their well-being such as life satisfaction and job satisfaction.

In fact, as reported in Table 1, guilt usually emerges in qualitative research but not in previous quantitative research.

### 1.1. Conflict, Guilt, Job Satisfaction and Life Satisfaction

Our study is based on the theory of social exchange, a theoretical framework focused on social relations in work contexts [45]. According to this theory, interpersonal connections work as a function of the rules of reciprocity [46]. The exchange process between work and family often occurs in stressful as well as entrepreneurial contexts [47] and impacts entrepreneurial well-being [48] and, consequently, the subjective success of the enterprise [49].

The role of the family in predicting life satisfaction seems to be fundamental for women entrepreneurs [9,50] because women continue to take on more family responsibility than men, and those responsibilities still remain when the woman has a career [51]. Role theory [52] has been used extensively to explain strategies for balancing family and working life. Let us assume a finite number of resources are available [53]. Based on the assumption that the greater the roles required, the greater the effort required to balance them, these roles move within a framework of conflict, sometimes called interference [25]. From this background, the incompatibility between the family domain and the work domain takes shape [54]. In general, conflict is negatively associated with the well-being of workers [55] and can affect both the personal and professional environment. In fact, since the different domains (family and work) require energy, time, dedication and commitment [56], the choice between one domain in favour of another generates not only negative effects, but also negative emotions such as frustration, shame, anger or guilt [57]. Guilt arises because past actions or behaviours conflict with internal moral standards or codes [58] and occurs when individuals feel that that they should have thought, felt or act in another way or the moral code is violated [59,60,61]. Conflict is associated with Guilt when there is a need to choose between work and family [62,63]. In particular, if the requests of the two domains (family and work) are incompatible, people choose work instead of family or vice versa, family instead of work [64]. This decision causes guilt [65] because the family is neglected because of work (Work Interference with Family, Guilt WIF) or work is neglected because of the family (Family Interference with Work, Guilt FIW). Some research has highlighted the interrelationship between the work–family conflict and the resulting Guilt [66,67]. With regard to women entrepreneurs specifically, some studies have highlighted the Guilt [34,68] over children or work. Research has often focused on job satisfaction and in particular on the negative effects of the work–family interface [55,69,70,71]. There is general agreement that the work–family conflict is related to job satisfaction [72]. In general, it can be postulated that when work–family conflict increases, job satisfaction decreases [55,73]. The meta-analysis by Kossek and Ozeki [71] showed a coherent negative relationship between negative conflict and job satisfaction More recent research has confirmed these findings [37,74,75]. The research has produced more and more results related to the influences of the work–family interface both in the family and in the workplace [55,76]. From the various research, it can be stated that a work–family interface perceived as negative leads to lower levels of psychological well-being than when the interface is positive [77]. Some initial studies took into consideration the one-dimensional work–family interface construct [78] by measuring its impact on family life [79]. Some studies have managed to highlight the moderating role of gender by indicating lower levels of well-being in women than in men [78]. Other subsequent studies have considered work–family conflict [75,80]. In general, research on conflict has shown that conflict decreases life satisfaction levels [81]. Some studies have considered the interrelation between work–family conflict and guilt [56,66,82,83] and found that family interference is positively associated with guilt. This guilt is in turn associated with different aspects of life such as life satisfaction [67]. Thus, situations such as conflict and guilt related to work and family, which arise from the need to make a choice, risk negatively interfering with life satisfaction and job satisfaction [84,85]. On the basis of these premises, this study proposes the following hypotheses:

**Hypothesis** **1** **(H1).** *Guilt (Work Interference with Family Guilt and Family interference with work Guilt) mediates the Conflict and Job Satisfaction relationship*.

**Hypothesis** **2** **(H2).** *Guilt (Work Interference with Family Guilt and Family interference with work Guilt) mediates the Conflict and Life Satisfaction relationship*.

### 1.2. The Moderating Role of Enrichment

To these previous considerations is added the concept of “enrichment”, which in contrast is based on the assumption that participation in multiple roles provides a greater number of resources, energies and expansion [86] applicable to other life domains [87]. Women entrepreneurs particularly benefited from effective work–family enrichment and family-derived enrichment [88]. The interface between work and family life has been studied extensively [80,89,90,91]. We believe it is appropriate to use the expression “work–family interface” because it does not exclude that the roles can be both positive and negative. By analysing the vast literature on this topic, in fact, it can be observed that the coexistence of work and family roles can be considered as positive or negative. In fact, some studies have highlighted the positive side of the work–family interface [76,92,93,94,95], embracing the idea of empowerment derived from the different social and psychological resources brought into play by multiple roles of life [19]. This approach assumes that participation in multiple roles is a source of opportunities and resources that leads to better functioning in other areas of life [87]. This generates individual positive effects, which have been defined as “positive spillover” [87,96], “enrichment” [53,64], and “facilitation” [76,92,93]. Few studies have instead considered the positive influences of family and work on professional satisfaction. Among them, some researchers found that positivity was related to job satisfaction [83,97,98]. Indeed, Hochwarter and colleagues [99] concluded that work-related guilt could be neutralized if people are able to manage resources at work, thereby increasing job satisfaction. Furthermore, it would seem that a work–family interface perceived as positive improves the quality of life [100] although the positive effects of the work–family interface have not yet been studied empirically in large measure [77,101]. The few studies conducted have showed that a positive family interface is associated with high levels of life satisfaction [70,93]. The work–family interface is also associated with guilt. Enrichment increases job satisfaction [83,87] and life satisfaction [70,97]. Greenhaus and Powell [94] hypothesized that enrichment could moderate the negative consequences of family conflict. Previous studies showed that Enrichment can buffer the negative effects of conflict [102]. However, most research in this area only verifies a simple additive relationship, with conflict and enrichment modelled as predictors. However, such additive models are only one of the many possible ways to conceptualize the work–family interface. On the basis of these premises, this study proposes the following hypotheses:

**Hypothesis** **3** **(H3).** *Enrichment moderates the indirect relationship between Conflict and Job Satisfaction via Guilt (Work Interference with Family Guilt and Family interference with work Guilt). Specifically, the indirect relationship between Conflict and Job Satisfaction is weaker for women entrepreneurs with high levels of enrichment*.

**Hypothesis** **4** **(H4).** *Enrichment moderates the indirect relationship between Conflict and Life Satisfaction via Guilt (Work Interference with Family Guilt and Family interference with work Guilt). Specifically, the indirect relationship between Conflict and Job Satisfaction is weaker for women entrepreneurs with high levels of enrichment*.

**Figure 1:**
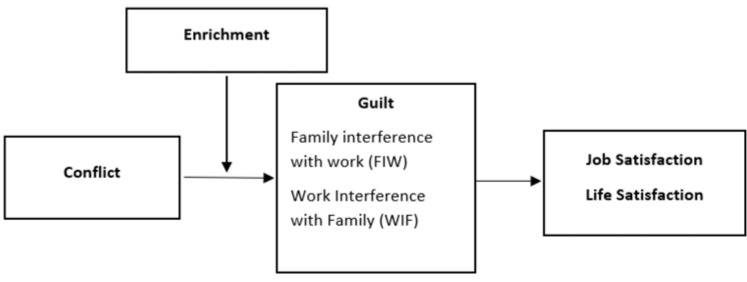
The proposed theoretical model.

Based on the above, we assume the model shown in Figure 1.

## 2. Materials and Methods

### 2.1. Participants and Procedure

The participants were recruited through some associations dedicated to entrepreneurs: AIDDA (Italian Association of Women Entrepreneurs and Corporate Executives) and Confindustria (General Confederation of Italian Industry). An anonymized questionnaire was presented to participants using two methods: online and on paper. There was no difference between the paper and online administration regarding the content and format of the questionnaire. The sample consisted of 161 women entrepreneurs over the age of 18. Participants were aged between 23 and 67 (M = 44.17; SD = 10.75); 7.7% of women entrepreneurs were involved in agricultural activities, 4.9% in crafts, 27.5% in the commercial sector, 3.5% in the construction sector, 13.4% in the restaurant/hotel sector and 43% in the service sector. The firms of the participants were mainly micro including up to 10 workers.

### 2.2. Measures

#### 2.2.1. Guilt

Guilt was assessed by “Work Family Guilt Scale” [103,104], whose translation accuracy for the Italian context has been verified through back translation. This scale is composed of 2 subscales. The first investigates Work Interference with Family Guilt (4 items; item example: “*I regret not being around for my family as much as I would like to*”). The second investigates Family Interference with Work Guilt (3 items; item example: “*I am worried about the quality of my work because I often put my family before my job*”). The entrepreneurs responded by expressing their degree of agreement with the claims on a 6-point Likert scale.

#### 2.2.2. Work–Family Interface

Conflict and enrichment dimensions were assessed by 14 items from Kinnunen and colleagues [105]. This instrument has been validated for the Italian context [106]. (Conflict item example: “*The demands of your job interfere with your home and family life*?”; Enrichment item example “*You manage your time at work more efficiently because at home you have to do that as well?*”). Response categories for all of the items ranged from 1 (‘never’) to 6 (‘very often’).

#### 2.2.3. Life Satisfaction

Satisfaction with life was assessed through the single item developed by Lance and colleagues [107]. Participants were requested to indicate their life satisfaction (item: *How satisfied are you with your life in general?*) on a 10-point rating scale ranging from ‘very dissatisfied’ (1) to ‘very satisfied’ (10).

#### 2.2.4. Job Satisfaction

Job satisfaction was measured using the Brief Overall Job Satisfaction [108]. Job satisfaction was measured with five items (item example: “*I really enjoy my work”*). The respondents evaluated their perceptions of satisfaction concerning their current job on a response scale from 1 to 6 (1 = strongly disagree, 6 = strongly agree).

For all the measures not validated for Italian context, the translation accuracy has been verified through back translation.

### 2.3. Data Analisys

Descriptive statistics for the variables of interest were first calculated, followed by zero order correlation analysis to determine the associations among these variables. The variance inflation factors were lower than the threshold level of 10, indicating that there was no multicollinearity problem [109]. To check for common method variance bias, we used Harman’s single-factor test [110]. No single factor explained most of the variance, so common method bias has not been to be a major problem in this study. The study examined whether the mediation process was moderated by enrichment. Moderated mediation is used to determine whether the magnitude of a mediation effect is conditional on the value of a moderator. To test our hypotheses, we followed the suggestions of Preacher et al. [111] and Hayes [112], first testing the main hypotheses (Hypotheses 1 and 2) using the PROCESS macro for SPSS (Model 4) created by Hayes [112]. Next, we incorporated the proposed moderator into the model as indicated by some authors [111,113] to test the moderate mediation model. Model 7 was used to test Hypotheses 3 and 4. For these analyses, predictor and interaction terms were mean centered. We used a bootstrapping method (5000 resamples) to test for the significance of the effects [114].

## 3. Results

### 3.1. Descriptive Statistics and Correlations

Table 2 indicates the means, standard deviations, reliability and correlation results for all variables of the study. The scale reliability was in the accepted range as the Cronbach’ Alpha was above 0.7 for all dimensions. Results of correlation show that Job Satisfaction and Life Satisfaction were positively associated with Enrichment (r = 0.259 and r = 0.631, respectively) and were negatively associated with Conflict (r = −0.271 and r = −0.513, respectively), Guilt WIF (r = −0.321 and r = −0.555, respectively) and Guilt FIW (r = −0.641 and r = −0.726, respectively).

### 3.2. Testing for Mediation Effect

The PROCESS macro for SPSS (Model 4) was used to test Hypothesis 1 and 2. Table 3 shows that Conflict had significant positive associations with Guilt FIW and Guilt WIF (t = 8.32, *p* < 0.001 and t =11,68 *p* < 0.001, respectively). When Conflict, Guilt FIW and Guilt WIF were entered into the regression equation with Job Satisfaction as the dependent variable, all predictors were negatively associated with Job Satisfaction. The mediation bootstrap confidence intervals did not contain zero (Conflict → FIW → Job satisfaction [−0.2153; −0.0425]; Conflict → WIF → Job satisfaction [−0.2241; −0.0067]. Therefore, Guilt FIW and Guilt WIF play a partial mediating role in the relationship between Conflict and Job Satisfaction. Thus, Hypothesis 1 was supported. When Conflict, Guilt FIW and Guilt WIF were entered into the regression equation with Life Satisfaction as a dependent variable, all predictors were negative associated with Life Satisfaction. The bootstrapped confidence intervals of the mediating association did not contain zero (Conflict → FIW → Life Satisfaction [−0.2952; −0.0592]; Conflict → WIF → Life Satisfaction [−0.3856; −0.1493]. Therefore, Guilt FIW and Guilt WIF conflict play a partial mediating role in the relationship between Conflict and Life Satisfaction. Thus, Hypothesis 2 was supported.

### 3.3. Moderated Mediation Effect Analysis

The PROCESS macro for SPSS (Model 7) was used to test Hypothesis 3 and 4. Results in Table 3 show the conditional process analysis. The findings further indicated that Enrichment has a direct significant negative effect on FIW and WIF (coeff. = −0.42590; *p* < 0.001 and coeff. = −0.25540; *p* < 0.0010, respectively). Additionally, the interaction of enrichment on the relationship between Conflict FIW and WIF appeared to be statistically significant (coeff. = −0.12960; *p* < 0.05 and coeff. = 40.09900; *p* < 0.0010; respectively). See Table 4.

**Figure 2:**
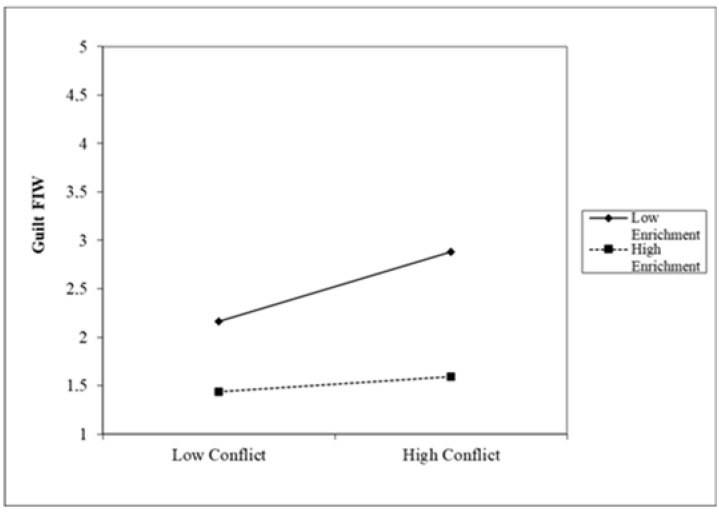
Moderating effect on Guilt FIW.

**Figure 3:**
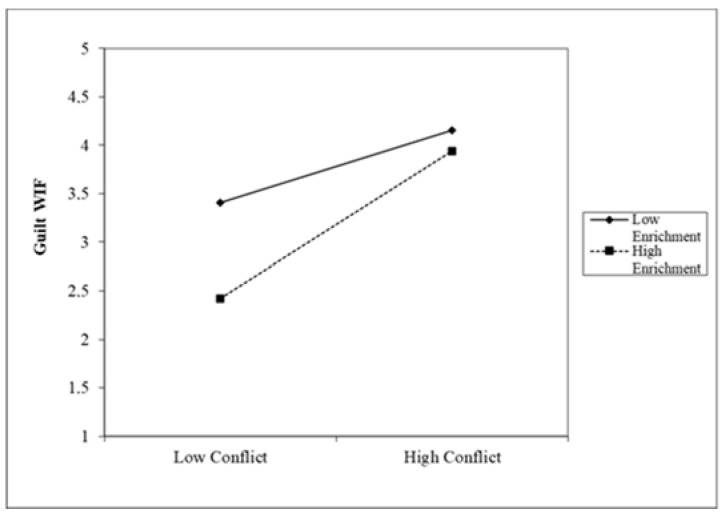
Moderating effect on Guilt WIF.

The interaction diagram directly reflects how the influence of Conflict on Guilt FIW and WIF was moderated by Enrichment (see Figure 2 and Figure 3). When the degree of Enrichment engagement was higher, the participants with high Conflict showed lower Guilt FIW and Guilt WIF, while the participants with low Enrichment showed higher Guilt FIW and Guilt WIF. In sum, moderated mediation models were established [115]. Guilt FIW and Guilt WIF mediates the relationship between work Conflict and Job satisfaction, as well as the relationship between Conflict and Life Satisfaction, and at the same time, Enrichment moderated the mediating processes by which the Conflict affects Job and Life Satisfaction via Guilt FIW and Guilt WIF. Based on these findings, Hypotheses 3 and 4 are therefore supported.

## 4. Discussion

This study aimed to analyse, first, two mediation models where Guilt FIW and Guilt WIF mediate the relationship between work Conflict and Job Satisfaction (Hypothesis 1), and the relationship between Conflict and Life Satisfaction (Hypothesis 2). Secondly we develop two models where higher levels of Enrichment are able to buffer these mediating processes by reducing the effects of Conflict on Job Satisfaction (Hypothesis 3) and Life Satisfaction (Hypothesis 4) via Guilt FIW and Guilt WIF. The results obtained allowed us to confirm all our Hypotheses. Indeed, Guilt FIW and Guilt WIF mediate the relationship between Conflict and Job Satisfaction, as well the relationship between Conflict and Life Satisfaction. Enrichment is able to moderate these mediating processes. Our results highlight the impact that Conflict and Guilt have on Job Satisfaction and Life Satisfaction [83,116]. This study helped fill the literature gap regarding women entrepreneurs and the work–family interface, including Guilt. It also took into account important factors that impact the quality of life such as Job Satisfaction and Life Satisfaction as outcomes. As stated by Hall [117], balancing work and family may represent a sacrifice of one domain for the other. Furthermore, women entrepreneurs mainly invest in the neoliberal market sacrificing the family for the business [118]. The research on the work–family interface in entrepreneurs has focused on the conflict and/or enhancement of the relationship between work and family domains [119,120,121,122]. The role pressures from family and work domains are seen as incompatible, as an inter-role conflict [54], even in entrepreneurship, but also as enrichment [88]. This study also showed the role of Enrichment in buffering the relationship between Conflict and Guilt, consistent with previous empirical findings. This can affect the beneficial effects of enrichment on both Life and Job Satisfaction in women entrepreneurs [41]. Is important to act to reduce the Guilt because an over-exposure to the feeling of Guilt may involve anxiety and depression [123]. This study has, of course, some limitations. First, it has used a cross-sectional design with self-reporting measures. Furthermore, it did not take into account the different characteristics of companies (size, years of activity, etc.). Our study was conducted in the Italian context, a country where the concept of Guilt is deeply rooted as are gender roles. It would be interesting in future studies to carry out a comparative study with other countries. 

Entrepreneurs play a fundamental role in the labour market because they are a real “driving force” [124] that cannot be eliminated Their entrepreneurial judgment allows them to anticipate future market trends and consequently to position their business [125]. Understanding the role of women entrepreneurs in the economy, beyond gender bias [126], is extremely important, as it would allow a less partial reading of economic, psychological and social phenomena. This study highlights the need to advance family friendly policies and improve affordable childcare services to for women entrepreneurs. In addition, in the economic field, recent research underlines the importance of public administrations in addressing the various issues of collective action [127]. Operationally, action should be taken to create contexts and services in which reconciling work and family [128,129] is easier for women entrepreneurs, also considering the implications relating to perceptions of entrepreneurial success [130].

## 5. Conclusions

The main objectives of this study were two. First, we sought to examine whether work interference with family guilt and family interference with work guilt would mediate the relationships between conflict and job satisfaction and between conflict and satisfaction in life. The second aim of the study was to test a moderate mediation model that could address both mediations (labor interference with family and family interference with work), highlighting the moderating role of enrichment. Results from the analysis support the hypothesized models. For these reasons, it is necessary to create protective contexts for women entrepreneurs. Therefore, implementing initiatives to support women entrepreneurs by implementing and diversifying specific family-friendly policies designed for them is critical.

## Figures and Tables

**Table 1:** Previous research.

Example References	Sample	Methodology	Focus
Kim, & Ling (2001) [17]; Teoh, et al. (2021) [35]; De Vita et al. (2019) [36]; De Clercq et al. (2021) [37].	Women Entrepreneurs	Quantitative	Conflict
Neneh, (2017) [38]; Sehgal, & Khandelwal, P. (2020) [39]; Welsh et al. (2018) [40]; İplik, & Ülbeği (2021) [41].	Women Entrepreneurs	Quantitative	Work-Family Interface, Enrichment
Ekinsmyth, C. (2014) [42]; Jamali (2009) [43]; Gill, & Ganesh (2007) [44]; De Simone & Priola, (2015) [32].	Mumpreneur, Women Entrepreneurs	Mixed/Qualitative	Studies in which Guilt emerges in entrepreneurship

**Table 2:** Means, standard deviation, reliability and correlations.

	M	SD	Reliability	1	2	3	4	5
1. Conflict	2.79	0.921	α = 0.840					
2. Guilt WIF	3.54	1.03	α = 0.738	0.273 **				
3. Guilt FIW	2.16	0.975	α = 0.832	0.583 ***	0.403 ***			
4. Enrichment	4.26	1.18	α = 0.879	−0.543 ***	−0.587 ***	−0.534 ***		
5. Job Satisfaction	3.44	0.955	α = 0.760	−0.271 ***	−0.321 ***	−0.641 ***	0.259 ***	
6. Life Satisfaction	7.34	0.885		−0.513 ***	−0.555 ***	−0.726 ***	0.631 ***	0.674 ***

Note. ** = *p* < 0.01 *** = *p* < 0.001; M = Mean SD = Standard Deviation.

**Table 3:** Mediation Analysis.

Regression Equation		Model			Significance of Regression Coefficients				
Outcome	Predictor(s)	R	R^2^	F	Coeff	t	LLCI	ULCI	*p*
Guilt FIW	Conflict	0.307	0.094	16.627	0.2839	8.3208	0.1464	0.4213	0.0001
Guilt WIF	Conflict	0.679	0.462	136.581	0.6364	11.6868	0.5289	0.7440	0.0000
Job Satisfaction	Conflict	0.743	0.552	64.636	−0.6144	−8.6509	−0.7547	−0.4741	0.0000
	Guilt FIW				−0.4266	−7.2701	−0.5425	−0.3107	0.0000
	Guilt WIF				−0.1728	−2.3041	0.3210	0.0247	0.0225
Life Satisfaction	Conflict	0.795	0.633	90.3906	−0.6018	−5.9685	−0.8010	−0.4026	0.0000
	Guilt FIW				−0.5884	−7.0625	−0.7529	−0.4238	0.0000
	Guilt WIF				−0.4132	−3.8796	−0.6235	−0.2028	0.0002

**Table 4:** Conditional process analysis.

Regression Equation		Model			Significance of Regression Coefficients				
Outcome	Predictors	R	R^2^	F	Coeff	t	LLCI	ULCI	*p*
Guilt FIW	Conflict	0.60	0.36	30.04	0.2359	2.6746	0.0617	0.4101	0.0083
	Enrichment				−0.4259	−6.3300	−0.5588	−0.2930	0.0000
	Int				−0.1296	−2.5739	−0.2291	−0.0302	0.0110
Guilt WIF	Conflict	0.73	0.54	61.49	0.6149	8.0749	0.4645	0.7653	0.0000
	Enrichment				−0.2554	−4.3967	−0.3701	−0.1407	0.0000
	Int				0.1782	4.0990	0.0923	0.2641	0.0001

## Data Availability

Restrictions apply to the availability of these data, which were used under permission for this study. The data are not publicly available due to privacy.
